# The Extracellular Matrix Influences Ovarian Carcinoma Cells’ Sensitivity to Cisplatinum: A First Step towards Personalized Medicine

**DOI:** 10.3390/cancers12051175

**Published:** 2020-05-07

**Authors:** Andrea Balduit, Chiara Agostinis, Alessandro Mangogna, Veronica Maggi, Gabriella Zito, Federico Romano, Andrea Romano, Rita Ceccherini, Gabriele Grassi, Serena Bonin, Deborah Bonazza, Fabrizio Zanconati, Giuseppe Ricci, Roberta Bulla

**Affiliations:** 1 Department of Life Sciences, University of Trieste, 34127 Trieste, Italy; abalduit@units.it (A.B.); alessandro.mangogna@studenti.units.it (A.M.); ggrassi@units.it (G.G.); rbulla@units.it (R.B.); 2 Institute for Maternal and Child Health, IRCCS Burlo Garofolo, 34134 Trieste, Italy; gabriella.zito@burlo.trieste.it (G.Z.); federico.romano@burlo.trieste.it (F.R.); giuseppe.ricci@burlo.trieste.it (G.R.); 3 Department of Medical, Surgical and Health Science, University of Trieste, 34129 Trieste, Italy; veronica.maggi@studenti.units.it (V.M.); andrea.romano@asuits.sanita.fvg.it (A.R.); sbonin@units.it (S.B.); deborah.bonazza@asuits.sanita.fvg.it (D.B.); fabrizio.zanconati@aots.sanita.fvg.it (F.Z.); 4 Centro Sociale Oncologico, OSARF, Azienda Sanitaria Universitaria Giuliano Isontina, 34127 Trieste, Italy; rita.ceccherini@asugi.sanita.fvg.it

**Keywords:** ovarian cancer, spheroids, chemoresistance, hyaluronic acid, fibronectin, personalized medicine, HGSOC

## Abstract

The development of personalized therapies for ovarian carcinoma patients is still hampered by several limitations, mainly the difficulty of predicting patients’ responses to chemotherapy in tumor cells isolated from peritoneal fluids. The main reason for the low predictive power of in vitro assays is related to the modification of the cancer cells’ phenotype induced by the culture conditions, which results in changes to the activation state and drug sensitivity of tumor cells compared to their in vivo properties. We have defined the optimal culture conditions to set up a prognostic test to predict high-grade serous ovarian carcinoma (HGSOC) patients’ responses to platinum chemotherapy. We evaluated the effects of hyaluronic acid (HA) and fibronectin matrices and the contribution of freezing/thawing processes to the cell response to platinum-based treatment, collecting spheroids from the ascitic fluids of 13 patients with stage II or III HGSOC. Our findings indicated that an efficient model used to generate predictive data for in vivo sensitivity to platinum is culturing fresh spheroids on HA, avoiding the use of previously frozen primary tumor cells. The establishment of this easy, reproducible and standardized testing method can significantly contribute to an improvement in therapeutic effectiveness, thus bringing the prospect of personalized therapy closer for ovarian carcinoma patients.

## 1. Introduction

Personalized medicine has gained a higher profile in oncology over the last decade, thanks to recent findings concerning the molecular and clinical characteristics of different tumors. Unfortunately, despite promising findings, the establishment of treatments specifically tailored to each patient is still hampered by several limitations, such as the substantial costs and lack of relevant models to mimic the patient.

The development of efficient personalized treatments for ovarian cancer patients is particularly worthwhile in relation to the high rate of chemoresistance occurrence. Indeed, aggressive front-line platinum-based chemotherapy, usually preceded by debulking surgery, is the cornerstone of treatment for ovarian carcinoma [1]; although it has an initial 80% cure rate, it frequently results in chemoresistance, which leads to recurrence within 18–24 months [2] and is responsible for a five-year survival rate below 45% [3].

One characteristic of ovarian cancer in comparison to other neoplasms is its tendency to form spheroids in the peritoneum, which create an isolated microenvironment. Ovarian cancer cells frequently disseminate and metastasize in the peritoneum, mainly due to the adherence of spheroids to the mesothelial layer of the peritoneum, the disaggregation and shedding of single cells from the spheroid core and the colonization of the extracellular matrix (ECM) of the mesothelial lining [4,5]. Spheroid budding from cell monolayers has also been frequently observed in culture, confirming their implication in dissemination [2]. In addition, ovarian carcinoma spheroids have been demonstrated to improve tumor resistance to treatments, protecting them from conventional chemotherapeutic agents and radiation-induced apoptosis [6].

In particular, many attempts have been made to predict the patient response to chemotherapy treatment through the establishment of cell cultures derived from liquid biopsies [7,8,9], albeit without obtaining truly satisfactory results. The main difficulty is the modification of the original phenotype of cancer cells by the culture conditions [10], resulting in changes in the activation state and drug sensitivity of the tumor cells as compared to their in vivo state. In this regard, several studies demonstrated the pivotal influence of culture media [11] and coating conditions on cancer cell modulation [12]. Moreover, particular attention has been paid to the role of ECM components in the in vitro modification of cancer cell hallmarks [5,13] and chemoresistance [14].

The aim of the current study was to define the appropriate culture conditions to accurately mimic the patient response to chemotherapy, in particular, with regard to the effects of the hyaluronic acid (HA) and fibronectin (FN) matrices. To this end, spheroids were collected from the ascitic fluids of 13 patients with stage II or III high-grade serous ovarian carcinoma (HGSOC) and characterized for platinum sensitivity. Furthermore, the contribution of the freezing/thawing process to the cell response to platinum-based treatment was analyzed.

## 2. Results

### 2.1. Hyaluronic Acid and Fibronectin Were Present in the Ovarian Carcinoma Stroma and Could Modulate Tumor Cell Behavior In Vitro in Response to Chemotherapeutic Treatment

**Figure 1:**
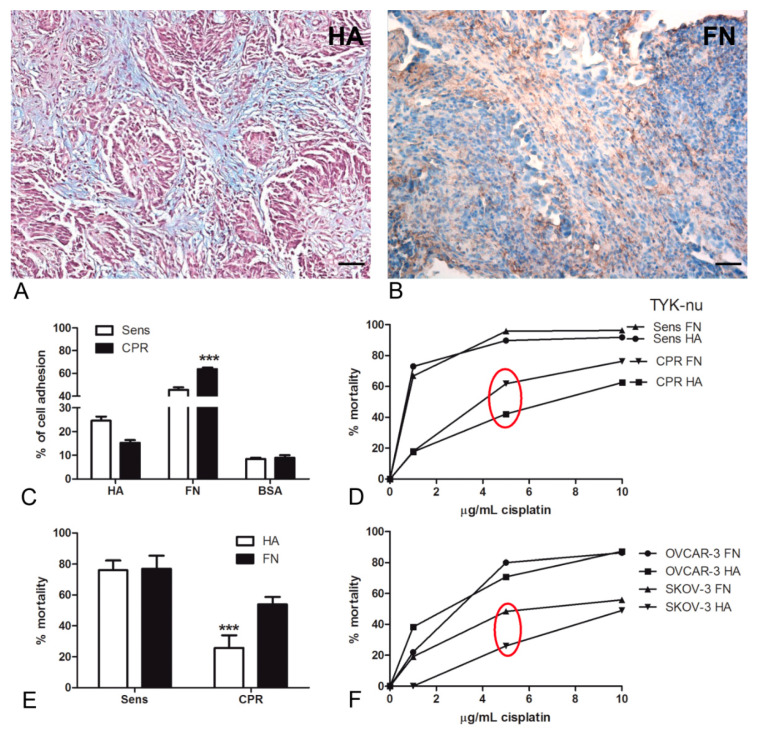
(**A**,**B**) Hyaluronic acid (HA) and fibronectin (FN) presence at the tissue level in human high-grade serous ovarian cancer (HGSOC). (**A**) Histochemical staining with Alcian Blue highlighted the HA distribution in HGSOC tissue sections; in particular, the staining was visible in tumor-associated stroma. Magnification, 200×; scale bar, 50 μm. (**B**) Immunohistochemical assay for the detection of FN at the tissue level showed a strong presence of FN in the tumor stroma and around blood vessels. A streptavidin–biotin–peroxidase system with 3,3′-Diaminobenzidine tetrahydrochloride (DAB) was used. Magnification, 200×; scale bar, 50 μm. (**C**–**E**) HA matrix maintained the chemoresistance of the ovarian cancer cell lines. Cisplatinum-sensitive (Sens) TYK-nu and cisplatinum-resistant (CPR) TYK-nu were stained with a fluorescent dye (Fast DiI) and seeded onto a HA- or FN-coated 96-well plate. The percentage of adhesion was interpolated using a calibration curve generated through the lysis of an increasing number of labeled cells. On HA, the adhesion of Sens TYK-nu was favored compared to that of CPR, whereas on FN, CPR appeared to be more adhesive than sensitive ones; both cell types preferentially adhered to FN. The fluorescence intensity was measured using Infinite200 (Tecan Group Ltd., Männedorf, Switzerland) (**C**). By evaluating the percentage of mortality after 72 h of treatment with 5 µg/mL of cisplatinum, we observed that hyaluronic acid was increasingly able to maintain chemoresistance as compared to FN. Cell viability was evaluated with an ELISA reader (O.D., 450 nm) (**D**,**E**). A killing assay was also performed with the OVCAR-3 and SKOV-3 cell lines, observing a similar behavior to that of the TYK-nu cells (**F**). *** *p* < 0.001.

We initially investigated the presence and the localization of HA and FN within the tumor microenvironment by histochemical and immunohistochemical analyses of HGSOC specimens. As shown in Figure 1A, Alcian Blue staining highlighted the presence of HA in the tumor stroma, whereas FN appeared to be abundantly present both in the stroma (Figure 1B) and around the vessels (Figure S1). The positivity of both these matrices co-localized with the tumor streak/grain in the tissue.

In order to achieve a more precise understanding of HA and FN involvement in modulating tumor behavior, we took advantage of TYK-nu, a human ovarian cancer cell line derived from an HGSOC patient [15]. In particular, we compared the cisplatinum-sensitive (Sens) TYK-nu to the cisplatinum-resistant (CPR) TYK-nu, obtained by culturing TYK-nu in the presence of cisplatinum in stepwise increasing concentrations [16]. First, we tested the capability of both cell types to interact with HA or FN through an adhesion assay (Figure 1C). We observed that with the addition of HA, the adhesion of platinum-sensitive cells was most favored (22% ± 5%) as compared to that of CPR cells (15% ± 5%). By contrast, on FN, platinum-resistant cells appeared to be more adhesive (63% ± 11%) than sensitive cells (45% ± 5%). Both cell types preferentially adhered to FN as compared to HA.

Subsequently, TYK-nu cells seeded on HA or FN were treated with different concentrations of cisplatinum. As shown in Figure 1D, 5 µg/mL of cisplatinum seemed to correspond to the main representative concentration for the IC50 value; at this concentration, the mortality of Sens TYK-nu appeared to be independent of matrix influence, whereas a statistically significant difference was observed in CPR cell lines (*p* < 0.001); CPR cells showed decreased mortality when seeded on HA (Figure 1E). In order to confirm these observations about chemoresistance, we repeated the killing assays using the OVCAR-3 and SKOV-3 cell lines; the latter are known to be resistant to platinum-based treatments. As indicated in Figure 1F, we noticed a similar trend: the cells seeded on HA showed decreased mortality as compared to those on FN. In particular, the most pronounced difference was once again observed in chemoresistant cells.

### 2.2. FN Was Able to Increase Cell Proliferation through MAPK Activation

**Figure 2:**
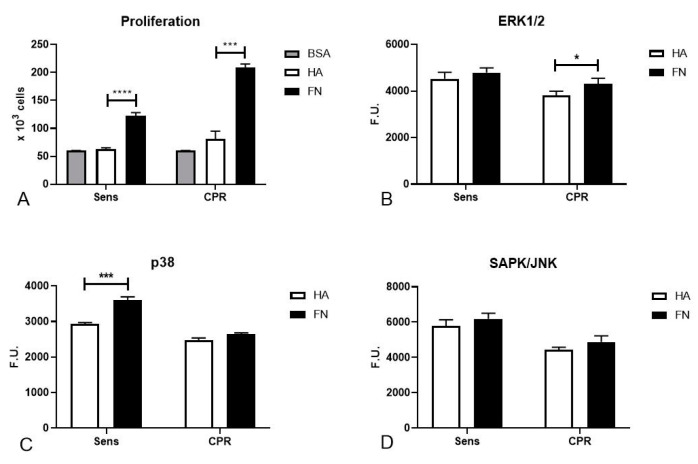
FN stimulation of proliferation in ovarian cancer cell lines. (**A**) TYK-nu cells, after overnight (ON) starvation, were seeded onto the HA or FN matrix in order to evaluate cell proliferation. Bovine serum albumin (BSA) was used as a negative control. FN seemed to significantly enhance cell proliferation. (**B**–**D**) Phosphorylation of ERK1/2, p38 and SAPK/JNK was evaluated in TYK-nu cells through a PathScan^®^ Intracellular Signaling Array kit. Cells were allowed to adhere to HA and FN for 20 min, and phosphorylation was measured in total lysates. A fluorescence readout was acquired and expressed as fluorescence units (F.U). using the LI-COR Biosciences Infrared Odyssey imaging system (Licor Biosciences, Lincoln, NE, USA), and the data were processed using the software Image Studio 5.0 (Licor Biosciences, Lincoln, NE, USA). * *p* < 0.05; *** *p* < 0.001; **** *p* < 0.0001.

Aiming for more precise knowledge of the mechanisms involved in the increased mortality of ovarian cancer cells seeded on FN, we performed a proliferation assay with TYK-nu cells. Both Sens and CPR TYK-nu were subjected to serum starvation overnight (ON) in order to synchronize the cell cycles, and then seeded onto HA or FN matrices to evaluate if the different coating conditions were able to provide a stimulus for cell proliferation. We noticed that the cells on FN were more active in terms of proliferation as compared to the ones seeded onto HA (Figure 2A).

Next, we sought to understand which pathways could play a fundamental role in the influence exerted by FN on cell cycle regulation and cell proliferation with a PathScan^®^ Intracellular Signaling Array kit (Cell Signaling Technology Inc., Danvers, MA, USA). ON-starved TYK-nu cells were allowed to adhere to HA or FN for 20 min in order to detect the activation of different signaling pathways by incubating the array slide with cell lysates ON at 4 °C. In particular, we focused on the activation of mitogen-activated protein kinase (MAPK) cascades. We observed an increased activation of p38 in Sens TYK-nu cells, as shown in Figure 2B, whereas CPR cells showed an increased activation of extracellular signal-regulated kinase 1/2 (ERK1/2), as shown in Figure 2C. Nevertheless, a statistically significant difference could not be discerned for stress-activated protein kinase (SAPK/JNK), as shown in Figure 2D.

### 2.3. FN’s Role in Regulating DNA Damage and Inducing Apoptosis in Platinum-Sensitive Cells

**Figure 3:**
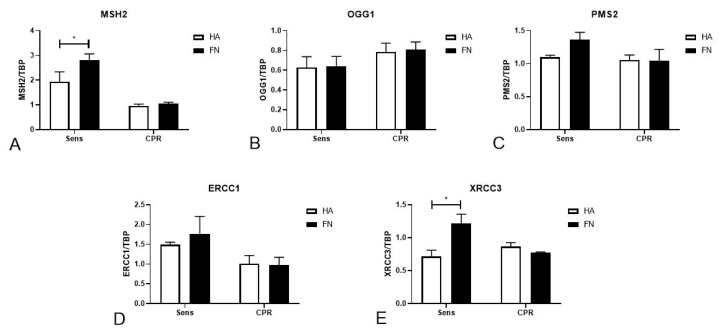
FN regulation of genes involved in DNA damage repair. TYK-nu cells were seeded onto a HA and FN matrix for 24 h; then, total RNA was extracted from cell lysates, and real-time qPCR was performed to evaluate the gene expression of MSH2 (**A**), OGG1 (**B**), PMS2 (**C**), ERCC1 (**D**) and XRCC3 (**E**). MSH2 and XRCC3 expression were significantly increased by FN’s influence, whereas HA seemed to downregulate their expression. TATA-box binding protein (TBP) was used as a housekeeping gene to normalize gene expression results. * *p* < 0.05.

In the attempt to deepen our insight into the molecular mechanisms that could play a fundamental role in the FN-mediated regulation of cell mortality after cisplatinum treatment, we evaluated the expression levels of five genes responsible for DNA repair after damage: MutS homolog 2 (MSH2), 8-oxoguanine glycosylase (OGG1), PMS1 homolog 2 (PMS2), excision repair cross-complementation group 1 (ERCC1) and X-ray repair cross complementing 3 (XRCC3). Gene expression was evaluated through real-time qPCR (Figure 3A–E), upon seeding TYK-nu cells onto a HA or FN matrix for 24 h. We observed that MSH2 and XRCC3 expression was significantly increased by FN’s influence, whereas HA seemed to downregulate their expression, as shown in Figure 3A,E. The other genes did not seem to be regulated by HA or FN stimuli.

**Figure 4:**
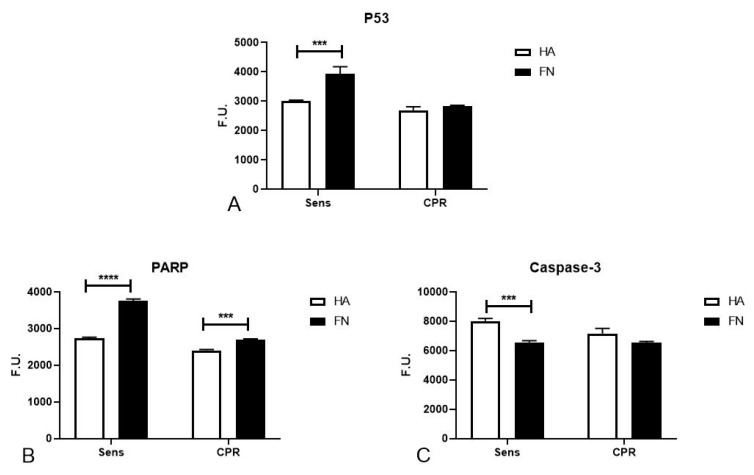
FN regulation of p53, PARP and caspase-3 activation. Phosphorylation of p53 (**A**), PARP (**B**) and caspase-3 (**C**) was evaluated in TYK-nu cells with a PathScan^®^ Intracellular Signaling Array kit. Cells were allowed to adhere to HA and FN for 20 min, and phosphorylation was measured in total lysates. A fluorescence readout was acquired using the LI-COR Biosciences Infrared Odyssey imaging system (Millennium Science), and the data were processed using the software Image Studio 5.0. *** *p* < 0.001; **** *p* < 0.0001.

The PathScan^®^ assay allowed us to highlight a potential role of FN in regulating p53 phosphorylation such as apoptosis pathway activation; in particular, we point out that in Sens TYK-nu, FN was able to significantly increase p53 activation, as shown in Figure 4A, and PARP activation (Figure 4B), while at the same time decreasing caspase-3 activation (Figure 4C).

### 2.4. Adhesion and Spheroid Formation in Culture

**Figure 5:**
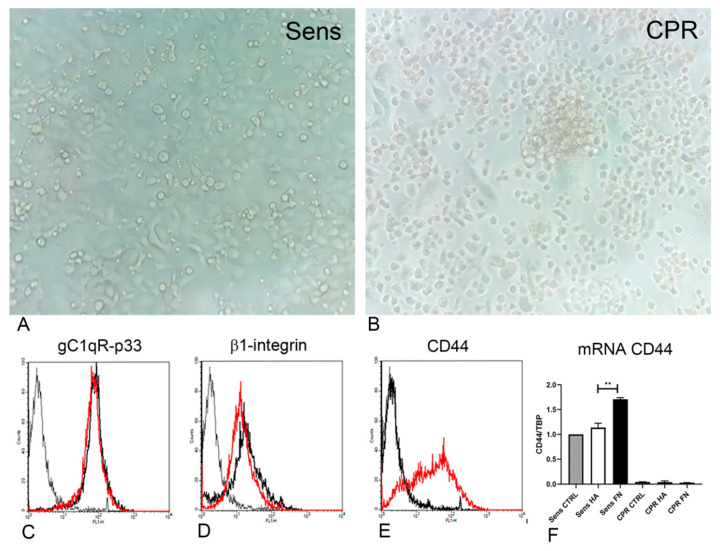
Sens and CPR TYK-nu showed different behaviors under shaking conditions. (**A**,**B**) Sens TYK-nu and CPR TYK-nu were seeded onto HA and FN matrices and allowed to grow into confluent tight monolayers. After 6 h on an orbital shaker (130 RPM), CPR TYK-nu underwent the formation of spheroids (**B**), whereas Sens TYK-nu maintained their original culture features. (**A**) Cells were evaluated under a Leica DMIL inverted microscope (Leica Microsystem, Milan, Italy), and images were collected using a Canon Powershot A640 digital camera (Canon, Tokyo, Japan). Original magnification: 200×. (**C**–**E**) A cytofluorimetric characterization of Sens (dashed line) and CPR (black line) TYK-nu was performed in terms of β1-integrin (**C**), HA binding protein (**D**)—also known as gC1qR/p33—and CD44 (**E**) expression. The fluorescence intensity of the cells incubated with primary antibodies was compared with unrelated straining (gray line). The expression of CD44 by CPR TYK-nu was completely absent. These data were also confirmed by evaluating CD44 mRNA expression level through real-time qPCR experiments (**F**), which highlighted an increased level of CD44 when the cells were seeded onto FN as compared to on HA. ** *p* < 0.01.

Observations of ovarian cancer cells’ capacity to agglomerate and form spheroids in culture were improved by evaluating the behavior of TYK-nu cells, both Sens and CPR, seeded onto different matrices. Once they reached a tight confluence as a monolayer, the cells seeded onto HA or FN were subjected to gentle shaking in order to mimic the movement of cells within the peritoneal liquid (Figure 5A,B). As a consequence of this treatment, CPR cells formed spheroids, nevertheless maintaining a consistent portion of adherent cells, as is visible in Figure 5B. By contrast, Sens TYK-nu maintained a monolayer organization (Figure 5A).

Both Sens and CPR TYK-nu cells were evaluated through FACS analysis in order to investigate the expression of adhesion receptors, such as β1-integrin, gC1qR/p33/hyaluronan binding protein and CD44. No differences could be highlighted as regard β1-integrin and gC1qR/p33/hyaluronan binding protein expression (Figure 5C,D).We observed a substantial difference in CD44 expression, since CPR cells showed a complete loss of CD44, as represented in Figure 5E. To achieve a clearer understanding of this result, we performed also a real-time qPCR using TYK-nu cells seeded onto HA or FN, in order to evaluate CD44 expression at the mRNA level. As shown in Figure 5F, the loss of CD44 expression in CPR TYK-nu was confirmed by this technique. Furthermore, we could see an increased level of CD44 expression when the cells were seeded onto FN as compared to HA (*p* < 0.01).

### 2.5. Primary Cells Isolated from Peritoneal Fluids of Ovarian Carcinoma Patients Presented Peculiar Characteristics and Culture Behavior

Having demonstrated that HA and FN have potential effects on the behavior of ovarian cancer cell lines, we proceeded with the evaluation of their roles in primary tumor cells isolated from patients. We obtained peritoneal fluids from 65 patients enrolled in the Obstetrics and Gynecology Unit of IRCCS Burlo Garofolo, Trieste, with suspicious ovary masses. In order to obtain a more consistent cohort, we had to carry out a selection of patients mainly based on tumor histotype and exposure to chemotherapeutic drugs, focusing our attention on 13 patients affected by HGSOC and undergoing cisplatinum treatment. As shown in Table 1, the mean age at diagnosis was 64.2 ± 7.1 years old. The vast majority of patients showed peritoneal carcinomatosis (92.3%) and ascites (69.2%) at clinical presentation; 23.1% of patients (*n* = 3) were germinal BRCA1 and/or BRCA2 mutated. In 30.8% (*n* = 4) of patients, the stage at diagnosis was I–II, while in 69.2% (*n* = 9), it was III–IV.

**Figure 6:**
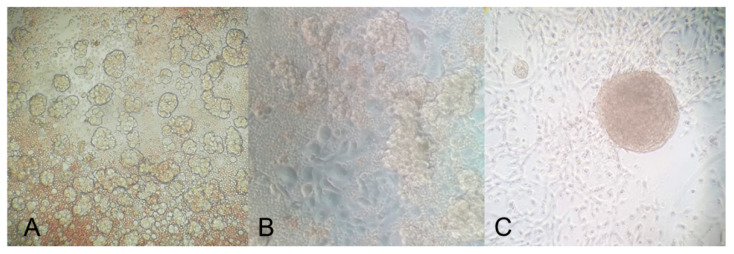
A few hours after cell isolation, a strong component of spheroids was visible in culture (**A**); after 24 h, although the presence of spheroids in suspension was still massive, adherent cells became predominant (**B**); after the first passage in culture, cells showed the capacity to agglomerate and return in suspension as spheroids, particularly when the cells were seeded onto the HA matrix (**C**). The mounted coverslips were examined under a Leica AF6500 microscope using the LAS software (Leica). Original magnification: 200×.

Primary cells were isolated from the peritoneal fluids or peritoneal washings of patients affected by ovarian carcinoma, as confirmed by the immunophenotypic characterization of the tumor mass at the moment of diagnosis (Table S1). As red blood cells could interfere with the adhesion process for single cancer cells and spheroids, we tried to remove as many erythrocytes as possible by lysis with specific reagents, taking care to avoid cell damage and to not activate nucleated cells. A few hours after isolation, the cells derived from peritoneal fluids revealed wide heterogeneity, including both adherent and floating cells as spheroids (Figure 6A). After 24 h, nevertheless, the presence of spheroids in suspension was still detectable; adherent cells became predominant and exhibited the characteristic morphology of epithelial cells, as shown in Figure 6B. After the first culture passage, some populations showed the capacity to agglomerate/aggregate independently and return in suspension as spheroids (Figure 6C), particularly when cells were grown on HA.

**Figure 7:**
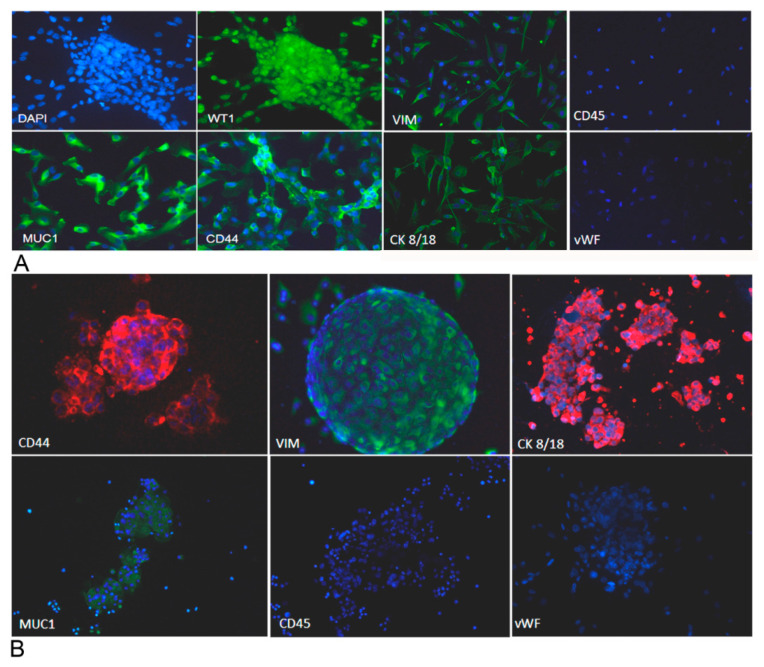
Ovarian cancer adherent cells and spheroids were characterized for the expression of different markers. We evaluated the expression of several markers for ovarian cancer cells by immunofluorescence, upon fixation and permeabilization. Adherent cells (**A**) appeared positively stained for vimentin, cytokeratin 8/18, mucin 1, CD44 and WT-1 and negatively stained for von Willebrand factor and CD45, excluding endothelial cell and blood cell contamination. Spheroids (**B**), after cytocentrifugation and fixation, were stained for vimentin, cytokeratin 8/18, mucin 1, CD44 and WT-1. Nuclei were stained with DAPI. Original magnification: 100×.

Adherent cells and spheroids, immediately after isolation, were characterized for the expression of several markers through an immunofluorescence assay. We evaluated the expression of mucin-1 (MUC1), cytokeratin 8/18 (CK 8/18) and Wilms’ tumor 1(WT-1), all diagnostic markers of ovarian cancer, and vimentin and CD44 were also analyzed. As expected, cells were positive for all the markers, as shown in Figure 7A,B. Cells were also stained to investigate the expression of the von Willebrand factor (vWF) and CD45, with the purpose of excluding contaminant endothelial cells or leukocytes.

### 2.6. Sensitivity to Cisplatinum Treatment Was Modified by the Different Matrix Cultures

**Figure 8:**
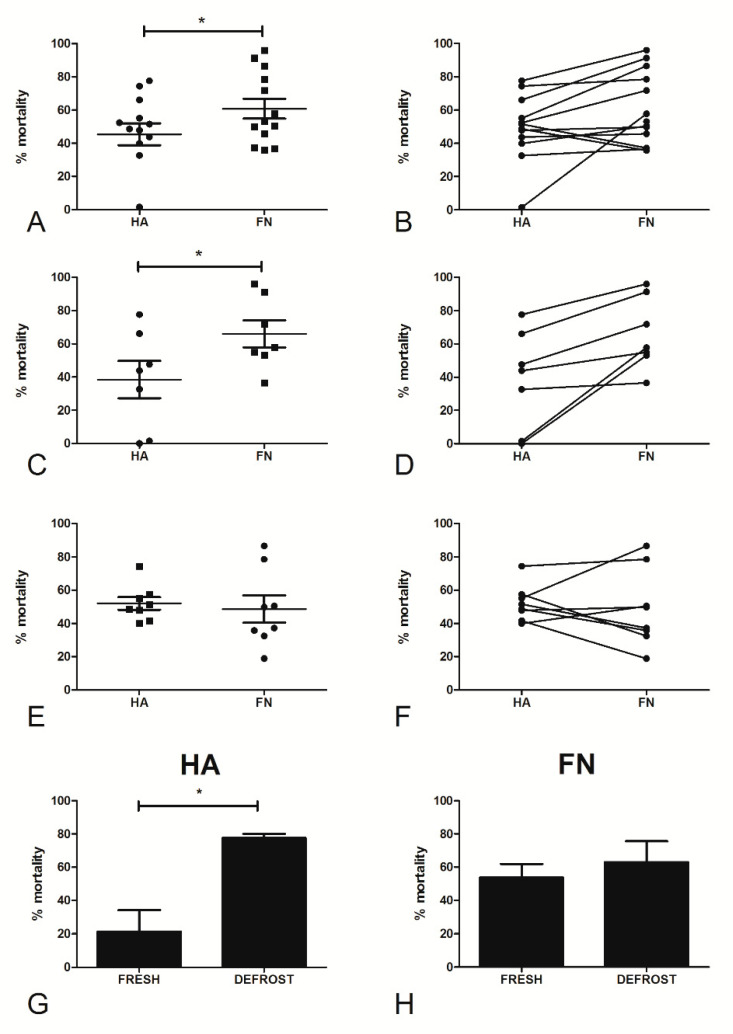
The effect of HA and FN matrices on the cisplatinum sensitivity of ovarian cancer primary cells. Fifteen different ovarian cancer cell populations, either fresh or after a freezing/thawing process, were seeded onto HA or FN matrices and allowed to adhere overnight; the following day, the cells were treated with 5 μg/mL of cisplatinum for 72 h. At the end of the incubation, WST-1 was added to determine the percentage of cell viability (**A**,**B**). When analyzing only fresh populations, we observed that the HA matrix was able to increase cell chemoresistance as compared to FN, in a statistically significant manner (**C**,**D**), whereas defrosted populations did not show statistical differences (**E**,**F**). With four ovarian cancer primary populations, we performed a killing assay on both fresh and frozen/thawed cells, seeded onto HA or FN matrices. Fresh cells seeded onto HA showed increased chemoresistance as compared to the same frozen/thawed populations (**G**). Instead, cells seeded onto FN demonstrated a similar percentage of mortality before and after the freezing/thawing process (**H**). * *p* < 0.05.

The primary aim of our study was to investigate how cells grown on different matrices modified their behavior in terms of sensitivity to cisplatinum treatment. In order to evaluate the influence of matrices on chemosensitivity, primary ovarian cancer cells isolated from patients with suspected HGSOC were seeded onto HA or FN and treated with different concentrations of cisplatinum. Having performed killing assays on cells isolated from all the samples collected between 2017 and 2019 (Figure S2), we included in the data analysis only 13 populations to select a consistent subset of patients, focusing on the ones affected by HGSOC and undergoing cisplatinum treatment. On the basis of the results obtained for the TYK-nu cell lines, we compared the percentage of mortality after treatment with 5 µg/mL cisplatinum; a statistically significant difference (*p* < 0.05) in terms of mortality was observed between cells seeded onto HA or FN (Figure 8A,B). Indeed, cells seeded onto FN appeared to be more sensitive to chemotherapeutic drugs, probably as a consequence of the pro-proliferative boost exerted by this matrix. As a result, we confirmed that HA was able to reinforce a status of chemoresistance to platinum, as previously observed in CPR TYK-nu.

As the eight cell populations encompassed in the present study were isolated, frozen and stored in liquid nitrogen for many months before proceeding with experiments, we decided to separate the data observed in frozen populations from those regarding fresh populations. By comparing Figure 8C,D and Figure 8E,F, a significant difference (*p* < 0.05) between cells cultured on HA or FN and treated with cisplatinum was seen only in fresh cells.

In order to confirm our hypothesis that the cryopreservation process was involved in the alteration of chemoresistance, we performed killing assays on four ovarian cancer cell populations, before and after freezing/thawing. We observed substantially different behavior between cells on HA and FN (Figure 8G,H). Whereas FN seemed not to be effective in affecting chemoresistance to cisplatinum before and after the freezing/thawing process, cells grown on HA showed a loss of chemoresistance after freezing/thawing (*p* < 0.05).

### 2.7. Ovarian Cancer Cells’ Culture Conditions Modified the Relationship between Cisplatinum-Induced Cell Mortality and the Patient Response to Chemotherapy

**Figure 9:**
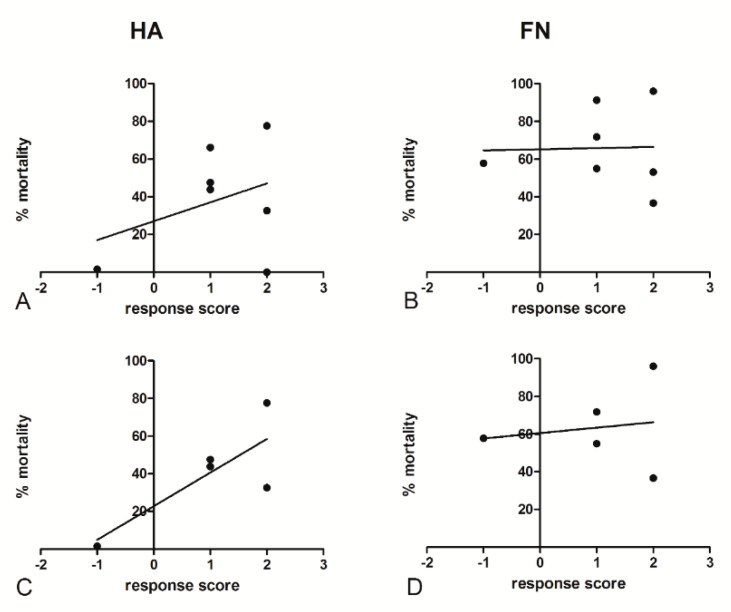
Analysis of the relationship between primary cells’ in vitro mortality and patients’ responses to chemotherapy. RECIST guideline parameters were associated to a response score after the first three cycles of cisplatin treatment: −2/−1, progressive disease (PD); 0, stable disease (SD); +1, partial response (PR); and +2, complete response (CR). No significant correlation was identified for the killing assays performed on the FN matrix (**B**), whereas those on HA showed a moderate correlation coefficient (**A**); the correlation coefficient was higher when BRCA-mutated patients were excluded (**C**). No significant correlation was identified for the killing assays performed on the FN matrix even when BRCA-mutated patients were excluded (**D**). Pearson’s test allowed us to perform a correlation assessment by taking into account the percentage of in vitro mortality after 5 μg/mL cisplatin treatment, both on HA and FN, and the clinical response score.

Evaluating the relationship between the in vitro response to chemotherapeutic drugs and clinical follow-up data of patients (Table 2) could be essential for the development of a potential strategy for precision medicine in ovarian cancer patients. To this end, we associated the Response Evaluation Criteria in Solid Tumors (RECIST) guideline to a response score, defined by considering the mass reduction after the first three cycles of cisplatin treatment: −2/−1 indicated progressive disease (PD); 0, stable disease (SD); +1, a partial response (PR); and +2, a complete response (CR) [17]. Pearson’s test allowed us to perform a correlation assessment by taking into account the percentage of in vitro mortality of ovarian cancer primary cells after 5 μg/mL cisplatin treatment, both on HA and FN matrices, and the clinical response score. The HA matrix seemed to potentially mimic the in vivo response to cisplatin treatment (Figure 9A), showing a moderate correlation coefficient (r = 0.360), whereas no significant correlation was identified for the killing assays performed on the FN matrix (Figure 9B). The correlation coefficient was moderately higher for the HA matrix (r = 0.796) when excluding BRCA-mutated patients (Figure 9C), which revealed divergent behaviors as compared to the other populations. A similar result could not be observed for the FN matrix (Figure 9D). In any case, the small cohort size hindered the achievement of a statistically significant *p*-value.

Having initially observed a complete loss of CD44 in CPR TYK-nu cells, we also investigated CD44 expression levels in primary cells by real-time qPCR, trying to correlate this information with the response score. In general, we noticed a higher level of CD44 expression when the primary cells were seeded onto FN (Figure S3), as previously reported for TYK-nu cells. In the end, it was not possible to find any correlation with the clinical data.

## 3. Discussion

The main challenge in establishing primary cell cultures from ovarian cancer peritoneal effusions is represented by the presence of free-floating cancer cells forming agglomerates as spheroids that often fail to attach to tissue culture substrata [18,19]; when they succeed, the pro-adhesive stimuli given by the matrices can completely modify the phenotypic characteristics of the original clones. The maintenance of the characteristics of tumor cells in a multicellular organization, to mimic the spheroids observed in the ascitic fluids of ovarian carcinoma patients, is made particularly complicated by the alteration of the cellular response due to the freezing procedure and culture passage [20,21]. The literature data reported the successful creation of several ovarian carcinoma cell lines [20,22,23], although often not taking into account the essential role of spheroids in ovarian cancer progression. It is necessary to bear in mind that established cell lines are an in vitro simplification of the tumor microenvironment and that heterogeneity is observed.

Among all the extracellular matrix components considered as possible culture substrata, we selected HA and FN, in agreement with Burleson and colleagues’ study, because they demonstrated a preferential adhesion of patient ascites’ spheroids to FN in comparison to type IV collagen and laminin. Furthermore, the authors observed a high percentage of primary tumor cell adhesion to HA. In addition, in our previous study, we investigated the role of HA in malignant pleural mesothelioma, analyzing its capability to modulate tumor growth by promoting cell adhesion and proliferation [24].

Although the presence of HA and FN in the ovarian carcinoma microenvironment was already suggested [25,26,27], we confirmed the evidence by histochemistry and immunohistochemistry. Both of these extracellular matrix molecules were abundantly present in the ovarian carcinoma tissue, particularly around neoplastic cells, indicating an active role in tumor development.

To get preliminary indications of the stimuli presented by HA and FN to tumor cells, we took advantage of the ovarian carcinoma cell line TYK-nu previously treated to become CPR, comparing it to Sens TYK-nu. Firstly, the adhesion assay data confirmed the binding of the ovarian cancer cell lines to the selected matrix components, showing a particularly preferential adhesion to FN. To enrich the information about adhesion, we also attempted to reach a deeper understanding of spheroid budding from monolayers by using TYK-nu cell lines and mimicking fluid shear stress, which was previously demonstrated as an essential physiological input for spheroid generation [28]. When Sens and CPR TYK-nu cells were grown in a dynamic culture, interestingly, we noticed budding from monolayers on both HA and FN, but only for CPR cells. This result was confirmed by previous reports addressing spheroids’ metastatic capacity [6] as well as their role in the acquisition of chemoresistance [29]. Spheroid formation could also be attributed to the complete disappearance of CD44 expression, as observed in CPR cells, inducing lower adhesion and leading to a reduction in the proliferation rate of these epithelial cells. Even if CD44’s involvement in ovarian cancer as a prognostic factor is still widely debated [30], our observations seem to be in accordance with the results published by Iseki et al. [31], who produced evidence that the loss of CD44 was associated with a reduced efficacy of chemotherapy; this was due to cell detachment from the basal membrane, as well as tumor cell invasion and metastasis.

We proceeded to investigate the influence of matrices in response to treatment with chemotherapeutic agents. The mortality of Sens cells appeared to be independent of coating conditions, whereas a statistically significant difference could be observed in CPR cell lines. HA, indeed, appeared to be able to reinforce resistance to platinum treatment. Similar behavior was noticed in SKOV-3, another chemoresistant cell line.

Having collected initial information on ovarian cancer cell lines, we moved towards the evaluation of primary cells from patients affected by HGSOC, in order to set up a translational study aimed at creating a prognostic test to predict patients’ responses to platinum chemotherapy treatment. We decided to use primary cells isolated from peritoneal fluids, avoiding the tumor mass, to make sample collection easier, reproducible and standardized. Despite enrolling a large number of initial patients (*n* = 65) with both ovarian malignancies and benign tumors, we carried out a selection process to obtain a more consistent cohort, focusing our study only on HGSOC patients undergoing cisplatinum treatment; this reduced the sample to 13 patients.

Once isolated, ovarian cancer cell populations appeared to be comprised of both single cells organized in adherent monolayers and cell agglomerates in suspension. We observed that primary cells grown on HA rather than on FN were more prone to undergoing the formation of aggregates in vitro, a process known as spheroid budding. Gong et al. [32] demonstrated that the treatment of ovarian cancer cell lines with FN, compared to with other ECM proteins, increased the levels of integrin expression in the spheroids of SKOV-3 and OVCAR-3 cells.

CPR TYK-nu cells’ resistance to platinum treatment was confirmed with primary ovarian cancer cells; cells cultured on HA were more resistant to the killing induced by cisplatin than those cultured on FN, maybe as a consequence of the pro-proliferative boost exerted by this ECM protein, already reported in multiple studies [33,34]. We highlighted FN’s capacity to increase cell proliferation by a proliferation assay, additionally supported by the PathScan^®^ assay results, which suggested that proliferation could be sustained by MAPK activation. In a research study by Matsuo and colleagues, FN was demonstrated to induce ERK1/2 and p38 activation in cancer, promoting cell invasion and proliferation [35]. According to our data, FN increased the activation of two different cascades of the MAPK pathway in Sens and CPR TYK-nu; in Sens TYK-nu cell lines, FN appeared to be more effective in the activation of the p38 signaling pathway, whereas in CPR cells, ERK1/2 was shown to be more active.

Furthermore, a research paper from Bragado et al. demonstrated that p53 is required for apoptosis induction through p38 MAPK activation [36]. Interestingly, cisplatinum’s mechanisms of action are connected to DNA damage recognition, p53 activation and apoptosis induction [37,38]. We suggested that FN stimulated DNA damage-induced apoptosis through the upregulation of the proteins responsible for DNA damage recognition and repair, such as MSH2, which participate in DNA damage signal transmission to downstream signaling cascades involving p53, MAPK and p73 [39]. FN also showed the capacity to increase p53 activation, which is well known for its capacity to trigger cell death by apoptosis but also nonapoptotic cell death through necrosis. It was already demonstrated that enhancing p53 levels sensitizes tumor cells to chemotherapy treatment and, in particular, to cisplatinum [40]. In addition, in Sens cells seeded on FN, we observed an increased level of PARP activation, by the DNA nick sensor enzyme, but lower caspase-3 activation, likely due to the absence of an ongoing apoptotic stimulus such as cisplatinum treatment. Based on our results, we could hypothesize that FN created an overall molecular environment conducive to DNA damage signals’ transmission and, consequently, to apoptosis, increasing cell death after platinum treatment. The PathScan^®^ assay did not allow us to clearly determine which pathways were proliferation-dependent or DNA repair-correlated.

Having initially included both fresh and defrosted ovarian cancer primary populations, at a later stage, we decided to consider them separately during the data processing. Our data strongly suggested that the matrix could be an essential player in the modification of responses to platinum treatment only when considering fresh cells. These results suggested that the freezing/thawing process could have completely changed the sensitivity to cisplatinum of the ovarian cancer primary cells. As other groups have highlighted the different behaviors of fresh and defrosted tumor cells [41,42,43,44], we systematically analyzed the same cell populations, fresh and defrosted, and observed that cells grown on HA showed a loss of chemoresistance after the freezing/thawing process, suggesting that such a process could have caused the depletion of a subpopulation responsible for the resistance to cisplatinum.

In order to evaluate which matrix component could be considered as an efficient model to mimic the tumor microenvironment and show a potential relationship with the patient response, we carefully analyzed clinical data from patients’ follow-up. We evaluated the radiological response after chemotherapy, including CA125 variation, by giving to each patient a response score value from −1 (PD) to +2 (CR). By Pearson’s test, a high correlation coefficient between the in vitro assays and clinical data could be found, in particular when excluding the data of BRCA-mutated patients, which showed different behavior in terms of platinum sensitivity, maybe due to the intrinsic characteristics of the “hot” tumor microenvironment of BRCA-mutated patients. In fact, this tumor microenvironment is defined by higher levels of inflammation, indicated by the presence of a large number of immune cells, such as higher levels of HLA-A [45] and more free and dysfunctional CD8+ T cells [46]. It is also known that an increased level of HA fragments is often associated with inflammatory states [47]. We suppose that this condition could have, somehow, determined the divergent behavior of the cells isolated from the BRCA-mutated patients. Although not obtaining a statistically significant correlation due to the limited number of patients, these data seemed to be in accordance with our hypothesis that culturing fresh spheroids on HA could represent a good model to potentially predict patients’ responses to chemotherapeutic agents, as compared to FN. In fact, FN-related stimuli to cell proliferation could, to some extent, create a microenvironment more sensitive to chemotherapeutic treatment. Despite the potential HA relationship with patients’ responses, if considering a larger cohort, this could result from the bias of a limited subset of patients; the main aim of our work remains to report the initial observations and representative results for the development of a simple model for personalized medicine, in order to predict chemoresistance to platinum treatment, but this may evolve, in the future, to a translational model to test new therapeutic targets.

## 4. Materials and Methods

### 4.1. Reagents and Antibodies

High molecular weight hyaluronic acid (HMW HA) was a kind gift from Professor Ivan Donati (Department of Life Sciences, University of Trieste, Italy). Fibronectin (FN) was purchased from Corning (Milan, Italy).

The following antibodies were used: mouse mAb anti-vimentin and mouse mAb anti-CD44, purchased from Sigma-Aldrich (St. Louis, MO, USA); rabbit anti-human vWF, rabbit anti-human CK8/18, rabbit anti-human FN and goat anti-mouse FITC-conjugated F(ab)’ from Dako (Milan, Italy); mouse mAb anti-human CD45-PE was bought from Immunotools (Friesoythe, Germany); rabbit anti-human MUC1 from Invitrogen (Monza, Italy); mouse anti-human CD44 was bought from Thermo Fisher Scientific (Milan, Italy); mouse anti-human β1-integrin from Merck Millipore (Darmstadt, Germania); mouse anti-human gC1qR was a kind gift from Professor Berhane Ghebrehiwet (Department of Medicine, State University of New York, Stony Brook, NY, USA); and FITC-conjugated goat anti-rabbit and Cy3-conjugated goat anti-rabbit were purchased from Jackson ImmunoResearch (Milan, Italy). All chemicals were purchased from Sigma-Aldrich.

### 4.2. Patients

Between 2017 and 2019, 65 patients who attended the department of Obstetrics and Gynecology of the Institute for Maternal and Child Health IRCCS Burlo Garofolo of Trieste (Italy) with a pelvic mass diagnosed by ultrasound were enrolled. After diagnosis, the initial cohort of epithelial and nonepithelial ovarian tumors was classified as follows: benign (*n* = 16), borderline (*n* = 5) and malignant (*n* = 44). Ovarian malignancies, in turn, were divided into: serous (*n* = 28), clear-cell (*n* = 4), endometrioid (*n* = 2), other nonepithelial histotypes (*n* = 3) or metastases of other tumors (*n* = 7). A total of 13 cases met all the criteria of the 2014 WHO Classification of Tumors of Female Reproductive Organs [48] for HGSOC and were included in the current study (Table 1), also on the basis of chemotherapeutic treatment. The patient staging was assigned according to the International Federation of Gynecology and Obstetrics (FIGO) parameters [49,50]. Patients signed an informed consent form, following the approval of the ethical considerations by the CEUR (protocol number 4829), the regional ethical committee for Friuli Venezia Giulia, Italy.

### 4.3. Immunohistochemical Analysis

Tissue samples of HGSOC were fixed in 10% buffered formalin, paraffin-embedded and stored at 4 °C. The paraffin was steadily removed and the sections were rehydrated by washing them in xylene, 100% EtOH, 95% EtOH, 70% EtOH and distilled water. Antigen retrieval was performed in Tris-HCl/EDTA buffer, pH 9, for 20 min at 95 °C. After the inhibition of endogenous peroxidases with H_2_O_2_ for 5 min, the sections were incubated with dPBS + 2% BSA + 0.4% casein + 0.1% gelatin for 30 min in order to block unspecific binding and then with anti-human FN (1:400) ON at 4 °C. Antibodies were detected using the Vectastain Elite ABC horseradish peroxidase (HRP) kit (Vector Laboratories, Milan, Italy), and secondary antibodies were revealed using the DAB kit (Dako). The sections were counterstained with hematoxylin (Dako).

### 4.4. Alcian Blue Staining

After deparaffinization and rehydration, the tissue sections were incubated with a solution of 1% Alcian Blue dissolved in 3% acetic acid, pH 2.5, for 30 min at room temperature (RT). After washing them in tap water for 10 min, the sections were dehydrated and mounted.

Slides were examined under a Leica DM 3000 optical microscope, and images were acquired using a Leica DFC320 digital camera (Leica Microsystems, Wetzlar, Germany).

### 4.5. Coating Conditions

Culture surfaces were incubated ON at 4 °C with HA or FN at a concentration of 50 µg/mL in 0.1 M bicarbonate buffer, pH 9.6.

### 4.6. Cell Isolation and Culture

Human ovarian cancer cells were isolated from the peritoneal fluids or peritoneal washings of patients undergoing laparoscopy or laparotomy at the Institute for Maternal and Child Health IRCCS Burlo Garofolo of Trieste, subsequent to a suspected pelvic mass.

Samples were filtered through a 300-µm cell strainer (PluriSelect, Leipzig, Germany), in order to remove macroscopic debris, and centrifuged at 250× *g* for 7 min. After counting, red blood cells (RBC) were removed with RBC Lysis Buffer (Roche, Sigma-Aldrich). The cells were seeded onto pre-coated 24-well plates and cultured with Human Endothelial Serum-Free Medium (Life Technologies, Carlsbad, CA, Italy) supplemented with 20 ng/mL of epidermal growth factor, 10 ng/mL of basic fibroblast growth factor (Immunological Sciences), 1% penicillin–streptomycin (Sigma-Aldrich) and 10% fetal bovine serum (FBS, Life Technologies). Ovarian carcinoma cells were maintained at 37 °C in a humidified atmosphere in a 5% *v*/*v* CO_2_ incubator.

Sens TYK-nu and CPR TYK-nu cell lines were kindly provided by the University of Turku, Finland, as part of the HERCULES consortium (project-hercules.eu) and were cultured in DMEM supplemented with 10% FBS. OVCAR-3 and SKOV-3 were kindly provided by Prof. Daniele Sblattero, University of Trieste, Italy, and cultured in HESFM and RPMI, respectively, supplemented with 10% FBS.

### 4.7. Immunofluorescence Microscopy of Ovarian Cancer Adherent Cells and Spheroids

Cells were seeded onto round glass coverslips of 15 mm diameter and allowed to grow up to 70% confluence. Cells were fixed with 3% paraformaldehyde (PFA) for 15 min and then incubated with a solution of 1% BSA, 0.1% Triton X-100 and 50 mM glycine in dPBS in order to perform blocking, permeabilization and quenching, for 30 min at RT. Primary antibodies diluted in dPBS + 2% BSA were added for 1 h, and secondary antibodies, for 30 min at RT. Nuclei were stained with DAPI. Glass slides were mounted with a Fluorescence Mounting Medium (Dako) and images were acquired with a Leica DM 3000 fluorescence microscope using a Leica DFC320 camera.

Spheroids in suspension were cytocentrifuged through Thermo Shandon Cytospin 3 (Thermo Scientific) at 500 RPM for 5 min. The slides were air-dried for 10 min and fixed with ice-cold acetone for 5 min. Then, the same procedure described for adherent cells was followed.

### 4.8. Adhesion Assay

The adhesion assay was performed as previously described [51]. Briefly, cells were resuspended in dPBS containing 10 μg/mL of the fluorescent dye FAST DiI (Molecular Probes, Invitrogen) and incubated for 15 min at 37 °C in a 5% *v*/*v* CO_2_ incubator. After removing the excess FAST DiI with dPBS, 10^5^ cells were resuspended into a medium containing 0.1% BSA and seeded in a 96-well plate. The cells were allowed to adhere for 35 min at 37 °C in a 5% *v*/*v* CO_2_ incubator and then washed once with dPBS containing 0.5% BSA, 0.7 mM Ca^2+^ and 0.7 mM Mg^2+^, in order to remove nonadherent cells. Adherent cells were lysed by adding a solution of 10 mM Tris HCl, pH 7.4, and 0.1% *v*/*v* SDS. The plate was immediately read with a Tecan fluorescence reader using a calibration curve generated through the lysis of an increasing number of labeled cells.

### 4.9. Proliferation Assay

Ovarian cancer cells were starved ON in DMEM + 0.5% BSA. The following day, 2 × 10^4^/well ovarian cancer cells, resuspended in DMEM + 0.5% BSA, were seeded onto a previously HA and FN-coated 96-well plate and allowed to adhere ON. Then, cells were counted with particle counter Coulter Z1 (Beckman, Mundelein, IL, USA). For counting, cells were diluted 1:10,000 in 0.9% NaCl and 1:25 lysis buffer.

### 4.10. Flow Cytometry

First, 5 × 10^5^ ovarian cancer cells were fixed in 3% PFA in the dark for 20 min; next, they were incubated with primary antibodies diluted in dPBS + 2% BSA, 0.7 mM CaCl_2_ and 0.7 mM MgCl_2_ for 45 min on ice. Incubation with FITC-conjugated secondary antibodies was performed for 30 min on ice in the dark. The cells were resuspended and fixed in 1% paraformaldehyde.

Fluorescence was acquired with the FACScalibur (BD Bioscience, San Jose, CA, USA), and the data processed using the CellQuest software.

### 4.11. Real-Time Quantitative PCR

Ovarian cancer cells were grown onto a previously HA- and FN-coated 24-well plate for 24 h at 37 °C in a 5 % *v*/*v* CO_2_ incubator. Total RNA was isolated with a total RNA kit (EuroClone, Milan, Italy) and retrotranscribed to cDNA with the SuperMix kit (Bioline, Meridian Life Science, Memphis, TN, USA). Gene expression was evaluated by comparative quantification based on reaction efficacy and normalized to the expression of TBP as a housekeeping gene. The expression level of the following genes was investigated: MSH2, OGG1, PMS2, ERCC1, XRCC3 and CD44. The primers’ sequences are reported in Table S2.

The reaction was performed using the Rotor-Gene 6000 (Corbett Research, Mortlake, Australia), following a program of 45 cycles of denaturation (60 s at 95 °C), annealing (30 s at 60 °C, the melting temperature of the primers) and amplification (60 s at 72 °C).

### 4.12. Pathway Analysis (PathScan^®^ Intracellular Signaling Array Kit)

Pathway analysis was carried out following the instructions for the PathScan^®^ Intracellular Signaling Array Kit (Cell Signaling Technology, EuroClone, Milan, Italy). In brief, cells were starved ON, and 1 × 10^6^ cells/well were allowed to adhere to a previously coated six-well plate, for 20 min at 37 °C in a 5% *v*/*v* CO_2_ incubator. After a washing step with ice-cold dPBS, cells were lysed in a Cell Lysis buffer containing a cocktail of protease inhibitors (Roche Diagnostics, Monza, Italy). An Array Blocking Buffer was added to each well and incubated for 15 min at RT. Subsequently, an equal amount of total lysate (0.6 mg/mL) was added to each well and incubated for 2 h at RT. After washing, the biotinylated detection antibody cocktail was added to each well and incubated for 1 h at RT. Streptavidin-conjugated DyLight 680 was added to each well and incubated for 30 min at RT. A fluorescence readout was acquired using the LI-COR Biosciences Infrared Odyssey imaging system (Millennium Science), and the data processed using the software Image Studio 5.0.

### 4.13. Killing Assay

Primary ovarian cancer or TYK-nu cells were seeded onto a 96-well plate at a concentration of 5 × 10^3^ cells/well. The cells were allowed to settle down ON and treated with a different concentration of cisplatin (1, 5 or 10 µg/mL) for 72 h at 37 °C in a 5% *v*/*v* CO_2_ incubator. The percentage of viability, calculated as compared to nontreated cells, was detected by adding the WST-1 reagent of the Quick Cell Proliferation Kit (Abcam, Cambridge, MA, USA) and reading the plate at 450 nm with a spectrophotometer.

To evaluate the correlation between the in vitro mortality and clinical data, a response score was created following the RECIST guidelines to measure the radiological response after chemotherapy: −2/−1 was associated with PD, 0 with SD, +1 with PR and +2 with CR [17]. The correlation was assessed with Pearson’s Chi-squared test.

### 4.14. Statistical Analysis

Data were analyzed using the GraphPad Prism software 5.0 (GraphPad Software Inc., La Jolla, CA, USA), carrying out paired two-tailed Student’s *t*-tests. The results are expressed as the mean ± SEM of experiments, performed in triplicate. *p*-values < 0.05 were considered statistically significant.

## 5. Conclusions

In conclusion, in the present study, we demonstrated that: (1) sensitivity to the cisplatinum-induced killing of ovarian cancer primary cells was modified by different ECM components; (2) the use of previously frozen primary tumor cells should be avoided; and (3) the cisplatinum-induced killing of fresh spheroids cultured on HA was correlated with the patient response to chemotherapy. Together, these findings represent a first step toward the development of an optimized in vitro method for the reliable evaluation of patient resistance to platinum treatment.

## Figures and Tables

**Table 1:** Features of patients and primary cells isolated from patients affected by HGSOC. Characteristics of the cohort of patients affected by HGSOC, enrolled in the study at the Department of Obstetrics and Gynecology of IRCCS Burlo Garofolo (*n* = 13). Cells isolated from the first six patients were frozen before using them for experiments. Values of CA-125 are expressed as UI/l. Notes: np = not present; stage: in ovarian cancer, stages are expressed in Roman numerals and each stage is further divided into A, B and C.

Patient Code	Age (Years)	Stage	Peritoneal Carcinosis	Ascites	Pleural Effusion	BRCA Germinal Mutation	CA125 Pre-Treatment (UI/L)
**1**	64	IIIC	+	+	-	no	490
**2**	51	IIIC	+	+	-	no	168
**3**	68	IIIC	+	+	-	yes	649
**4**	58	IIIC	+	+	-	no	108
**5**	64	IIIC	+	+	+	no	1116
**6**	58	IIIC	+	+	+	np	118
**7**	76	IIB	+	+	+	np	7172
**8**	71	IIIA	+	np	-	np	20
**9**	55	IIIA	+	-	-	yes	290
**10**	73	IIB	+	+	-	no	1232
**11**	67	IIB	-	-	-	yes	16
**12**	69	IIIC	+	+	-	np	3175
**13**	60	IIB	+	-	-	np	70

**Table 2:** Clinical follow-up data of patients enrolled in the study. For each patient, pre- and post-treatment CA125 values, response scores following Response Evaluation Criteria in Solid Tumors (RECIST) guideline parameters and % of killing after in vitro cisplatinum-based treatment were reported. Values of CA-125 are expressed as UI/l. Notes: np = not present; CDDP = cisplatinum.

Patient Code	CA125 Pre-Treatment	CA125 Post-Treatment (3rd Cycle)	CA125 Post-Treatment (End)	Response Score	% Killing (CDDP 5 μg/mL)	
					FN	HA
**1**	490	16	278	−1	49.80	47.90
**2**	168	6	<35	2	50.47	39.99
**3**	649	171	<35	1	78.52	74.40
**4**	108	23	<35	2	86.51	55.11
**5**	1116	32	<35	2	35.79	48.64
**6**	118	45	<35	0	37.17	51.51
**7**	7172	457	45	1	45.76	43.81
**8**	20	np	<35	2	96.06	77.62
**9**	290	13	8	1	91.30	66.16
**10**	1232	625.9		−1	57.76	1.55
**11**	16	np	8.3	2	53.14	0.00
**12**	3175	43	58	1	71.83	52.40
**13**	70	6.5	5.6	2	36.61	32.60

## Supplementary Materials

The following are available online at https://www.mdpi.com/2072-6694/12/5/1175/s1, Figure S1: FN distribution around blood vessels in HGSOC tumor microenvironment. Figure S2: Effect of HA and FN matrixes on cisplatinum sensitivity of a mixed population of ovarian malignancies and benign tumors. Figure S3: CD44 mRNA expression levels in ovarian cancer cells isolated from ascitic fluids. Table S1: Immune-phenotypic characterization of the tumor mass. Table S2: Primers used for quantiative Real-Time PCR.

## References

[1] Kipps E., Tan D.S., Kaye S.B. (2013). Meeting the challenge of ascites in ovarian cancer: New avenues for therapy and research. Nat. Rev. Cancer.

[2] Ushijima K. (2010). Treatment for recurrent ovarian cancer-at first relapse. J. Oncol..

[3] Webb P.M., Jordan S.J. (2017). Epidemiology of epithelial ovarian cancer. Best Pract. Res. Clin. Obstet. Gynaecol..

[4] Lengyel E. (2010). Ovarian cancer development and metastasis. Am. J. Pathol..

[5] Burleson K.M., Casey R.C., Skubitz K.M., Pambuccian S.E., Oegema T.R., Skubitz A.P. (2004). Ovarian carcinoma ascites spheroids adhere to extracellular matrix components and mesothelial cell monolayers. Gynecol. Oncol..

[6] Al Habyan S., Kalos C., Szymborski J., McCaffrey L. (2018). Multicellular detachment generates metastatic spheroids during intra-abdominal dissemination in epithelial ovarian cancer. Oncogene.

[7] Mehta G., Hsiao A.Y., Ingram M., Luker G.D., Takayama S. (2012). Opportunities and challenges for use of tumor spheroids as models to test drug delivery and efficacy. J. Control. Release.

[8] Mulholland T., McAllister M., Patek S., Flint D., Underwood M., Sim A., Edwards J., Zagnoni M. (2018). Drug screening of biopsy-derived spheroids using a self-generated microfluidic concentration gradient. Sci. Rep..

[9] Hu L., McArthur C., Jaffe R.B. (2010). Ovarian cancer stem-like side-population cells are tumourigenic and chemoresistant. Br. J. Cancer.

[10] Miserocchi G., Mercatali L., Liverani C., De Vita A., Spadazzi C., Pieri F., Bongiovanni A., Recine F., Amadori D., Ibrahim T. (2017). Management and potentialities of primary cancer cultures in preclinical and translational studies. J. Transl. Med..

[11] Ackermann T., Tardito S. (2019). Cell Culture Medium Formulation and Its Implications in Cancer Metabolism. Trends Cancer.

[12] Liberio M.S., Sadowski M.C., Soekmadji C., Davis R.A., Nelson C.C. (2014). Differential effects of tissue culture coating substrates on prostate cancer cell adherence, morphology and behavior. PLoS ONE.

[13] Chen H., Nalbantoglu J. (2014). Ring cell migration assay identifies distinct effects of extracellular matrix proteins on cancer cell migration. BMC Res. Notes.

[14] Cho A., Howell V.M., Colvin E.K. (2015). The Extracellular Matrix in Epithelial Ovarian Cancer—A Piece of a Puzzle. Front. Oncol..

[15] Yoshiya N. (1986). Establishment of a cell line from human ovarian cancer (undifferentiated carcinoma of FIGO classification) and analysis of its cell-biological characteristics and sensitivity to anticancer drugs. Nihon Sanka Fujinka Gakkai Zasshi.

[16] Yoshiya N., Adachi S., Misawa Y., Yuzawa H., Honda T., Kanazawa K., Takeuchi S.T.K., Tanaka K. (1989). Isolation of cisplatin-resistant subline from human ovarian cancer cell line and analysis of its cell-biological characteristics. Nihon Sanka Fujinka Gakkai Zasshi.

[17] Schwartz L.H., Litiere S., de Vries E., Ford R., Gwyther S., Mandrekar S., Shankar L., Bogaerts J., Chen A., Dancey J. (2016). RECIST 1.1-Update and clarification: From the RECIST committee. Eur. J. Cancer.

[18] Verschraegen C.F., Hu W., Du Y., Mendoza J., Early J., Deavers M., Freedman R.S., Bast R.C., Kudelka A.P., Kavanagh J.J. (2003). Establishment and characterization of cancer cell cultures and xenografts derived from primary or metastatic Mullerian cancers. Clin. Cancer Res..

[19] Niedbala M.J., Crickard K., Bernacki R.J. (1985). Interactions of human ovarian tumor cells with human mesothelial cells grown on extracellular matrix. An in vitro model system for studying tumor cell adhesion and invasion. Exp. Cell Res..

[20] Allen H.J., Porter C., Gamarra M., Piver M.S., Johnson E.A. (1987). Isolation and morphologic characterization of human ovarian carcinoma cell clusters present in effusions. Exp. Cell Biol..

[21] Woods L.K., Morgan R.T., Quinn L.A., Moore G.E., Semple T.U., Stedman K.E. (1979). Comparison of four new cell lines from patients with adenocarcinoma of the ovary. Cancer Res..

[22] Ghani F.I., Dendo K., Watanabe R., Yamada K., Yoshimatsu Y., Yugawa T., Nakahara T., Tanaka K., Yoshida H., Yoshida M. (2019). An Ex-Vivo Culture System of Ovarian Cancer Faithfully Recapitulating the Pathological Features of Primary Tumors. Cells.

[23] Kar R., Chawla D., Gupta B., Mehndiratta M., Wadhwa N., Agarwal R. (2017). Establishment of Primary Cell Culture From Ascitic Fluid and Solid Tumor Obtained From Epithelial Ovarian Carcinoma Patients. Int. J. Gynecol. Cancer.

[24] Agostinis C., Vidergar R., Belmonte B., Mangogna A., Amadio L., Geri P., Borelli V., Zanconati F., Tedesco F., Confalonieri M. (2017). Complement Protein C1q Binds to Hyaluronic Acid in the Malignant Pleural Mesothelioma Microenvironment and Promotes Tumor Growth. Front. Immunol..

[25] Menzin A.W., Loret de Mola J.R., Bilker W.B., Wheeler J.E., Rubin S.C., Feinberg R.F. (1998). Identification of oncofetal fibronectin in patients with advanced epithelial ovarian cancer: Detection in ascitic fluid and localization to primary sites and metastatic implants. Cancer.

[26] Varankar S.S., More M., Abraham A., Pansare K., Kumar B., Narayanan N.J., Jolly M.K., Mali A.M., Bapat S.A. (2019). Functional balance between Tcf21-Slug defines cellular plasticity and migratory modalities in high grade serous ovarian cancer cell lines. Carcinogenesis.

[27] Kujawa K.A., Zembala-Nożyńska E., Cortez A.J., Kujawa T., Kupryjańczyk J., Lisowska K.M. (2020). Fibronectin and Periostin as Prognostic Markers in Ovarian Cancer. Cells.

[28] Masiello T., Dhall A., Hemachandra L.P.M., Tokranova N., Melendez J.A., Castracane J. (2018). A Dynamic Culture Method to Produce Ovarian Cancer Spheroids under Physiologically-Relevant Shear Stress. Cells.

[29] Liao J., Qian F., Tchabo N., Mhawech-Fauceglia P., Beck A., Qian Z., Wang X., Huss W.J., Lele S.B., Morrison C.D. (2014). Ovarian cancer spheroid cells with stem cell-like properties contribute to tumor generation, metastasis and chemotherapy resistance through hypoxia-resistant metabolism. PLoS ONE.

[30] Zhou J., Du Y., Lu Y., Luan B., Xu C., Yu Y., Zhao H. (2019). CD44 Expression Predicts Prognosis of Ovarian Cancer Patients Through Promoting Epithelial-Mesenchymal Transition (EMT) by Regulating Snail, ZEB1, and Caveolin-1. Front. Oncol..

[31] Iseki Y., Shibutani M., Maeda K., Nagahara H., Ikeya T., Hirakawa K. (2017). Significance of E-cadherin and CD44 expression in patients with unresectable metastatic colorectal cancer. Oncol. Lett..

[32] Gong L., Zheng Y., Liu S., Peng Z. (2018). Fibronectin Regulates the Dynamic Formation of Ovarian Cancer Multicellular Aggregates and the Expression of Integrin Receptors. Asian Pac. J. Cancer Prev..

[33] Han S.W., Roman J. (2006). Fibronectin induces cell proliferation and inhibits apoptosis in human bronchial epithelial cells: Pro-oncogenic effects mediated by PI3-kinase and NF-kappa B. Oncogene.

[34] Ahmed N., Riley C., Rice G., Quinn M. (2005). Role of integrin receptors for fibronectin, collagen and laminin in the regulation of ovarian carcinoma functions in response to a matrix microenvironment. Clin. Exp. Metastasis.

[35] Matsuo M., Sakurai H., Ueno Y., Ohtani O., Saiki I. (2006). Activation of MEK/ERK and PI3K/Akt pathways by fibronectin requires integrin alphav-mediated ADAM activity in hepatocellular carcinoma: A novel functional target for gefitinib. Cancer Sci..

[36] Bragado P., Armesilla A., Silva A., Porras A. (2007). Apoptosis by cisplatin requires p53 mediated p38alpha MAPK activation through ROS generation. Apoptosis.

[37] Di Pietro A., Koster R., Boersma-van Eck W., Dam W.A., Mulder N.H., Gietema J.A., de Vries E.G., de Jong S. (2012). Pro- and anti-apoptotic effects of p53 in cisplatin-treated human testicular cancer are cell context-dependent. Cell Cycle.

[38] Fuertes M.A., Castilla J., Alonso C., Perez J.M. (2003). Cisplatin biochemical mechanism of action: From cytotoxicity to induction of cell death through interconnections between apoptotic and necrotic pathways. Curr. Med. Chem..

[39] Tanida S., Mizoshita T., Ozeki K., Tsukamoto H., Kamiya T., Kataoka H., Sakamuro D., Joh T. (2012). Mechanisms of Cisplatin-Induced Apoptosis and of Cisplatin Sensitivity: Potential of BIN1 to Act as a Potent Predictor of Cisplatin Sensitivity in Gastric Cancer Treatment. Int. J. Surg. Oncol..

[40] Guntur V.P., Waldrep J.C., Guo J.J., Selting K., Dhand R. (2010). Increasing p53 protein sensitizes non-small cell lung cancer to paclitaxel and cisplatin in vitro. Anticancer Res..

[41] McDermott M., Eustace A.J., Busschots S., Breen L., Crown J., Clynes M., O’Donovan N., Stordal B. (2014). In vitro Development of Chemotherapy and Targeted Therapy Drug-Resistant Cancer Cell Lines: A Practical Guide with Case Studies. Front. Oncol..

[42] Aldridge B.B., Burke J.M., Lauffenburger D.A., Sorger P.K. (2006). Physicochemical modelling of cell signalling pathways. Nat. Cell Biol..

[43] Brigulova K., Cervinka M., Tosner J., Sedlakova I. (2010). Chemoresistance testing of human ovarian cancer cells and its in vitro model. Toxicol. Vitr..

[44] Le Gallo M., de la Motte Rouge T., Poissonnier A., Lavoue V., Tas P., Leveque J., Godey F., Legembre P. (2018). Tumor analysis: Freeze-thawing cycle of triple-negative breast cancer cells alters tumor CD24/CD44 profiles and the percentage of tumor-infiltrating immune cells. BMC Res. Notes.

[45] Menon A.G., Morreau H., Tollenaar R.A., Alphenaar E., Van Puijenbroek M., Putter H., Janssen-Van Rhijn C.M., Van De Velde C.J., Fleuren G.J., Kuppen P.J. (2002). Down-regulation of HLA-A expression correlates with a better prognosis in colorectal cancer patients. Lab. Investig..

[46] Trujillo J.A., Sweis R.F., Bao R., Luke J.J. (2018). T Cell-Inflamed versus Non-T Cell-Inflamed Tumors: A Conceptual Framework for Cancer Immunotherapy Drug Development and Combination Therapy Selection. Cancer Immunol. Res..

[47] Schwertfeger K.L., Cowman M.K., Telmer P.G., Turley E.A., McCarthy J.B. (2015). Hyaluronan, Inflammation, and Breast Cancer Progression. Front. Immunol..

[48] Kurman R.J., International Agency for Research on Cancer, World Health Organization (2014). WHO Classification of Tumours of Female Reproductive Organs.

[49] Berek J.S., Kehoe S.T., Kumar L., Friedlander M. (2018). Cancer of the ovary, fallopian tube, and peritoneum. Int. J. Gynaecol. Obstet..

[50] Prat J., Oncology F.C.o.G. (2014). Staging classification for cancer of the ovary, fallopian tube, and peritoneum. Int. J. Gynaecol. Obstet..

[51] Vidergar R., Agostinis C., Zacchi P., Mangogna A., Bossi F., Zanconati F., Confalonieri M., Ricci G., Bulla R. (2019). Evaluation of the Interplay Between the Complement Protein C1q and Hyaluronic Acid in Promoting Cell Adhesion. J. Vis. Exp..

