# Supplementary material for: The Extracellular Matrix Influences Ovarian Carcinoma Cells’ Sensitivity to Cisplatinum: A First Step towards Personalized Medicine
Source: Cancers (Basel). 2020 May 7;12(5):1175. doi: 10.3390/cancers12051175 (PMC7281165; doi:10.3390/cancers12051175)
Supplement: Supplementary file 1 [file cancers-12-01175-s001.pdf]

Supplementary Materials

The Extracellular Matrix Influences Ovarian Carcinoma Cells' Sensitivity to Cisplatinum: A First Step towards Personalized Medicine

Andrea Balduit, Chiara Agostinis, Alessandro Mangogna, Veronica Maggi, Gabriella Zito, Federico Romano, Andrea Romano, Rita Ceccherini, Gabriele Grassi, Serena Bonin, Deborah Bonazza, Fabrizio Zanconati, Giuseppe Ricci and Roberta Bulla

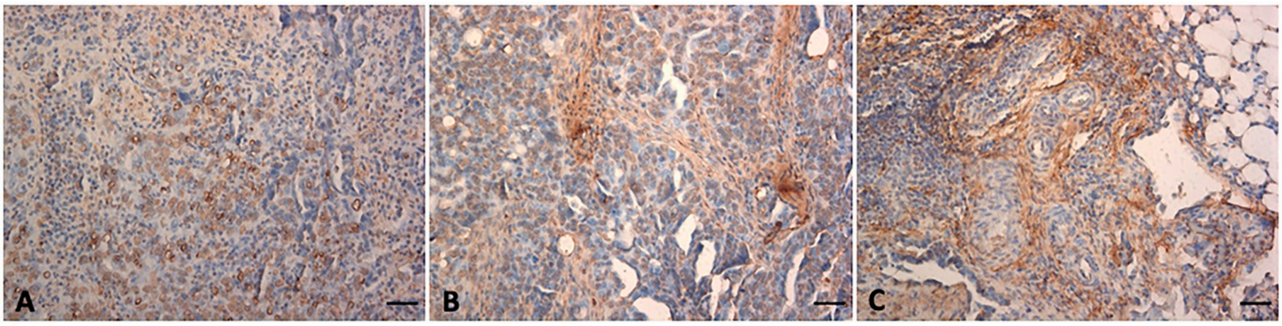

Figure S1. FN distribution around blood vessels in HGSOC tumor microenvironment. Magnification 200×, scale bar 50 μm.

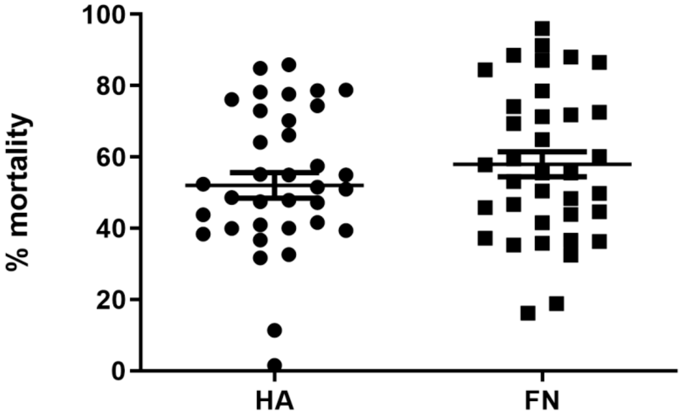

Figure S2. Effect of HA and FN matrixes on cisplatinum sensitivity of a mixed population of ovarian malignancies and benign tumors. In this graph, mortality after 5 μg/mL cisplatinum treatment was reported, by comparing HA and FN.

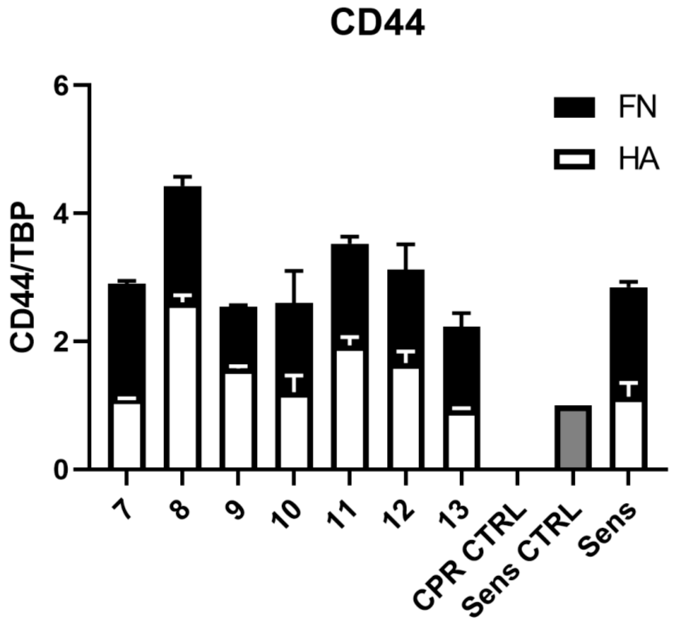

Figure S3. CD44 mRNA expression levels in ovarian cancer cells isolated from ascitic fluids. Cells were seeded onto HA and FN matrixes for 24 h. Total RNA was extracted from cell lysates and quantitative Real-Time PCR was performed to evaluate gene expression of CD44- TATA-box binding protein (TBP) was used as a housekeeping gene to normalize gene expression results. Sens TYK-nu were used as a calibrator.

Table S1. Immune-phenotypic characterization of the tumor mass. Notes: np = not present; PAX = paxillin; CK =cytokeratin; WT1 = Wilms' tumor 1; ER = estrogen receptor.

Patient Code	ki67 (%)	p53	PAX8	CK7	CK20	GATA3	WT1	ER
**1**	np	np	np	np	np	np	np	np
**2**	np	-	+	np	np	np	np	np
**3**	60	++	+	+	-	-	-	+
**4**	np	++	+	+	-	-	-	np
**5**	np	++	++	++	-	-	++	np
**6**	np	np	+	+	np	np	np	-
**7**	70	++	+	+	-	-	+	np
**8**	50	++	+	np	np	-	np	+
**9**	np	np	np	np	np	np	np	np
**10**	60-70	+++	++	+++	-	-	+	+++
**11**	np	+++	+	+	-	-	+	+++
**12**	np	-	+	+	np	np	+	np
**13**	np	++	+	+	-	-	+	++

Table S2. Primers used for quantiative Real-Time PCR.

Gene	Sequence (5' → 3')	Tm (°C)	NCBI GeneID
**MSH2**	AGGCATCCAAGGAGAATGATTG<br>GGAATCCACATACCCAACTCCAA	60	4436
**ERCC1**	TTTGGCGACGTAATTCCCGAC<br>CCTGCTGGGGATCTTTCACA	61	2067
**OGG1**	ACTCCCACTTCCAAGAGGTG<br>GGATGAGCCGAGGTCCAAAAG	60	4968

**PMS2**	CAATGGATGTGGGGTAGAAGAAG<br>GTTAGGTCGGCAAACTCTTGAAT	60	5395
**XRCC3**	CCCCATTCCGCTGTGAATTTG<br>GGTTAGCCCAGGTTATGCCA	61	7517
**CD44**	CTGCCGCTTTGCAGGTGTA<br>CATTGTGGGCAAGGTGCTATT	60	960
**TBP**	GAGCCAAGAGTGAAGAACAGTC<br>GCTCCCCACCATATTCTGAATCT	60	CR456776.1

© 2020 by the authors. Licensee MDPI, Basel, Switzerland. This article is an open access article distributed under the terms and conditions of the Creative Commons Attribution (CC BY) license (http://creativecommons.org/licenses/by/4.0/).
